# Resveratrol Partially Prevents Rotenone-Induced Neurotoxicity in Dopaminergic SH-SY5Y Cells through Induction of Heme Oxygenase-1 Dependent Autophagy

**DOI:** 10.3390/ijms15011625

**Published:** 2014-01-22

**Authors:** Tsu-Kung Lin, Shang-Der Chen, Yao-Chung Chuang, Hung-Yu Lin, Chi-Ren Huang, Jiin-Haur Chuang, Pei-Wen Wang, Sheng-Teng Huang, Mao-Meng Tiao, Jin-Bor Chen, Chia-Wei Liou

**Affiliations:** 1 Department of Neurology, Kaohsiung Chang Gung Memorial Hospital and Chang Gung University College of Medicine, Kaohsiung 833, Taiwan; E-Mails: tklin@cgmh.org.tw (T.-K.L.); jp1916@ms4.hinet.net (S.-D.C.); ycchuang@adm.cgmh.org.tw (Y.-C.C.); linhungyu700218@gmail.com (H.-Y.L.); suika68@cgmh.org.tw (C.-R.H.); 2 Mitochondrial Research Unit, Kaohsiung Chang Gung Memorial Hospital and Chang Gung University College of Medicine, Kaohsiung 833, Taiwan; E-Mail: jhchuang@adm.cgmh.org.tw (J.-H.C.); shenteng@adm.cgmh.org.tw (S.-T.H.); tmm@adm.cgmh.org.tw (M.-M.T.); chenjb1019@gmail.com (J.-B.C.); 3 Center of Parkinson’s Disease, Kaohsiung Chang Gung Memorial Hospital and Chang Gung University College of Medicine, Kaohsiung 833, Taiwan; 4 Department of Biological Sciences, National Sun Yat-Sen University, Kaohsiung 804, Taiwan; 5 Division of Pediatric Surgery, Kaohsiung Chang Gung Memorial Hospital and Chang Gung University College of Medicine, Kaohsiung 833, Taiwan; 6 Department of Internal Medicine, Kaohsiung Chang Gung Memorial Hospital and Chang Gung University College of Medicine, Kaohsiung 833, Taiwan; 7 Department of Chinese Medicine, Kaohsiung Chang Gung Memorial Hospital and Chang Gung University College of Medicine, Kaohsiung 833, Taiwan; 8 Department of Pediatrics, Kaohsiung Chang Gung Memorial Hospital and Chang Gung University College of Medicine, Kaohsiung 833, Taiwan; 9 Department of Nephrology, Kaohsiung Chang Gung Memorial Hospital and Chang Gung University College of Medicine, Kaohsiung 833, Taiwan

**Keywords:** Parkinson’s disease, oxidative stress, mitochondrial dysfunction, autophagy, apoptosis, resveratrol, heme oxygenase-1

## Abstract

Parkinson disease (PD) is a complex neurodegenerative disorder characterized by a progressive loss of dopaminergic neurons. Mitochondrial dysfunction, oxidative stress or protein misfolding and aggregation may underlie this process. Autophagy is an intracellular catabolic mechanism responsible for protein degradation and recycling of damaged proteins and cytoplasmic organelles. Autophagic dysfunction may hasten the progression of neuronal degeneration. In this study, resveratrol promoted autophagic flux and protected dopaminergic neurons against rotenone-induced apoptosis. In an *in vivo* PD model, rotenone induced loss of dopaminergic neurons, increased oxidation of mitochondrial proteins and promoted autophagic vesicle development in brain tissue. The natural phytoalexin resveratrol prevented rotenone-induced neuronal apoptosis *in vitro*, and this pro-survival effect was abolished by an autophagic inhibitor. Although both rotenone and resveratrol promoted LC3-II accumulation, autophagic flux was inhibited by rotenone and augmented by resveratrol. Further, rotenone reduced heme oxygenase-1 (HO-1) expression, whereas resveratrol increased HO-1 expression. Pharmacological inhibition of HO-1 abolished resveratrol-mediated autophagy and neuroprotection. Notably, the effects of a pharmacological inducer of HO-1 were similar to those of resveratrol, and protected against rotenone-induced cell death in an autophagy-dependent manner, validating the hypothesis of HO-1 dependent autophagy in preventing neuronal death in the *in vitro* PD model. Collectively, our findings suggest that resveratrol induces HO-1 expression and prevents dopaminergic cell death by regulating autophagic flux; thus protecting against rotenone-induced neuronal apoptosis.

## 1. Introduction

Parkinson’s disease (PD) is the second most common neurodegenerative disease in the world, affecting approximately 1% of adults older than 60 years [1]. Neuronal loss in the substantia nigra pars compacta (SNc) and the subsequent loss of striatal dopamine content are responsible for the classical motor features of PD [1–3]. However, many non dopaminergic nuclei are also affected in PD, including the locus coeruleus, reticular formation of the brain stem, raphe nucleus, dorsal motor nucleus of the vagus, basal nucleus of Meynert, amygdala, and hippocampus [1,3,4]. PD patients have a 2- to 5-fold higher risk of mortality than the general population [5,6]. Increasing evidence suggests that PD may be associated with mitochondrial dysfunction through a variety of pathways, including free-radical generation, inflammation, and mitochondrial respiratory chain complex I dysfunction [7–9]. Mitochondrial complex I dysfunction and oxidative stress play a crucial role in the pathogenesis of PD [7,9–12]. Additionally, mitochondrial respiratory chain dysfunction can eventually lead to both apoptotic and necrotic neuronal cell death [13–15].

Rotenone, which readily crosses the blood-brain barrier and accumulates throughout the brain, impairs mitochondrial function, ultimately leading to neurodegeneration [16,17]. Several biochemical abnormalities, including mitochondrial dysfunction and oxidative stress, thought to be relevant to the pathogenesis of PD were found in the brain of patients with PD [18]. Emerging evidence has suggested that mitochondrial dysfunction, increased oxidative stress, excitotoxicity, inflammation [19] and ubiquitin-proteasome system dysfunction may be involved in alpha-synuclein aggregation, Lewy body formation and neurodegeneration [18,20,21]. Furthermore, impaired mitochondria-specific autophagy, which is associated with a number of genes linked to PD, results in mitochondrial dysfunction and may directly induce neuronal dysfunction and neurodegeneration [22,23].

Plant phytoalexinsare generated *de novo* in response to infectious pathogens and increased disease resistance [24]. The natural phytoalexin, resveratrol (3,5,4′-*trans*-trihydroxystilbene), is a polyphenolic stilbene present in grapes and red wine. It possesses a variety of biological activities including anti-inflammatory and antioxidative activities [25,26]. A previous study in our laboratory, using a rat model of cholestasis (mediated by bile-duct ligation), demonstrated that resveratrol protects against liver cell apoptosis and induces autophagy [27]. Notably, a number of studies suggest that the protective effects of resveratrol are associated with the induction of autophagy [27–30].

The autophagy-lysosome pathway is responsible for intracellular clearance of misfolded proteins and prevents toxic accumulation of misfolded proteins and damaged organelles, which may threaten cell survival. It is an intracellular, catabolic process where proteins and organelles are isolated by the autophagosome, a double-membraned vesicle. The autophagosome subsequently fuses with endosomes to form hybrid organelles called amphisomes that, in turn, later fuse with lysosomes, where the entrapped cytosolic contents are digested and recycled [31,32]. Autophagy is thought to be important in protein-misfolding disorders such as PD. Enhancement of this process may provide a potential therapeutic strategy for neurodegenerative diseases.

Heme oxygenase (HO) is an enzyme that degrades intracellular heme to free iron, carbon monoxide and biliverdin [33]. Heme oxygenase-1 (HO-1) expression is induced not only by its physiological substrate heme but also by a wide variety of noxious stimuli or conditions, such as hyperoxia, hypoxia, proinflammatory cytokines, nitric oxide, heavy metals, UV irradiation, heat shock, shear stress, H_2_O_2_, and thiol-reactive substances [34]. HO-1 is rapidly induced by oxidative challenge and other noxious stimuli in the brain and in other tissues [35,36]. In addition, increased HO-1 levels in serum or within the substantia nigra of PD patients may indicate a systemic antioxidant reaction related to a state of chronic oxidative stress [37–39].

Here, we use an *in vitro* rotenone-induced PD model to verify the neuroprotective effects of resveratrol. Rotenone caused autophagosome accumulation, inhibition of HO-1 expression, and neuronal-cell apoptosis. Resveratrol acted in a neuroprotective manner to increase both HO-1 expression and autophagic flux with no effect on cell viability. Importantly, resveratrol partially rescued rotenone-induced apoptosis through a HO-1-associated increase in autophagy.

## 2. Results and Discussion

### 2.1. Resveratrol Prevents Rotenone-Induced Apoptosis in an Autophagy-Dependent Manner

**Figure 1.**
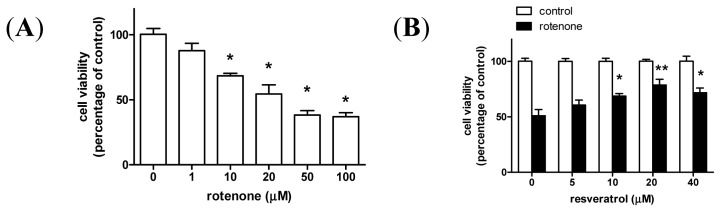

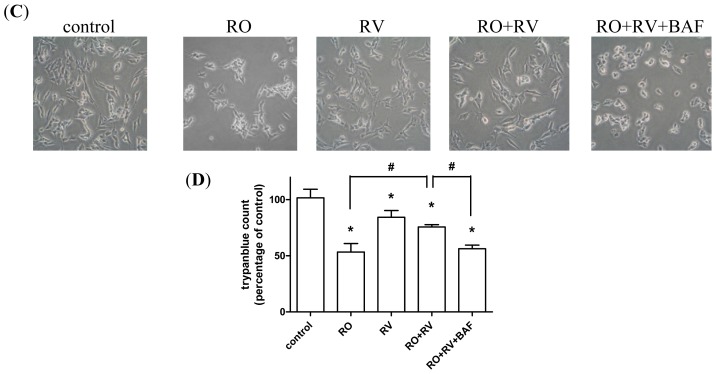
Resveratrol prevents rotenone-induced dopaminergic cell death in an autophagy-dependent manner. (**A**) SH-SY5Y cells were treated with various concentrations of rotenone (0–100 μM) for 24 h; (**B**) Cells were pretreated with various doses of resveratrol for 24 h, and then subjected to 20 μM rotenone for the next 24 h. Cell viability was determined by WST-8 assay using spectrophotometry at 450 nm; (**C**,**D**) Cells were pretreated with/without 20 μM resveratrol for 24 h, and then treated with/without 20 μM rotenone in the presence/absence of 10 nM bafilomycin A1 for the next 24 h. Cell viability was determined by light microscopy (magnified at 200×), and by counting trypan blue-stained cells. Cell counts are presented as a percentage of the control. RO, rotenone; RV, resveratrol; BAF, bafilomycin A1. Data (mean ± SEM) are representative of at least three independent experiments. Statistical significance was determined by one-way analysis of variance followed by Bonferroni post-hoc test. ^#^ *p* < 0.05 compared between groups. * *p* < 0.05 and ** *p* < 0.01 compared to control.

**Figure 2.**
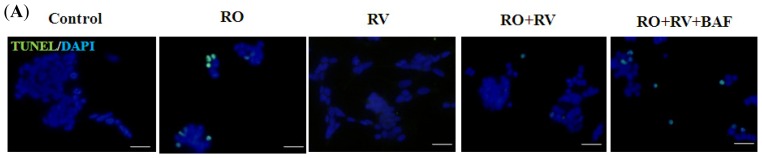

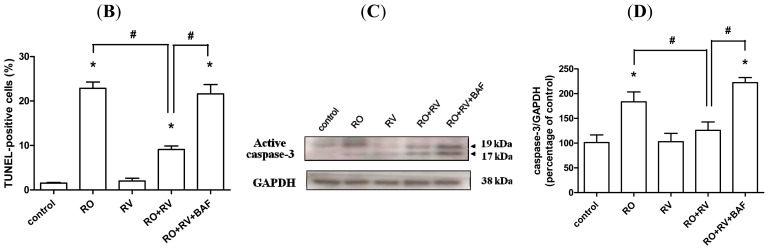
The protective effect of resveratrol against rotenone-induced neurotoxic apoptosis is associated with autophagy. SH-SY5Y cells pretreated with/without 20 μM resveratrol for 24 h were treated with/without 20 μM rotenone in the presence/absence of 10 nM bafilomycin A1 for the next 24 h. (**A**,**B**) Apoptotic cell death was detected by TUNEL immunofluorescence staining (green). Nuclei were stained with DAPI (blue). Scale bar, 50 μm. Apoptotic cells were quantitated using Image J (NIH, USA) and data are presented as the percentage of TUNEL-positive cells to DAPI-positive cells; (**C**,**D**) Immunoblots for the active (cleaved) form of caspase-3 and glyceraldehyde-3-phosphate dehydrogenase (GAPDH), which are a marker of apoptosis and loading control, respectively. Blots were analyzed using Image J. Data were normalized to GAPDH and expressed as a percentage of control. RO, rotenone; RV, resveratrol; BAF, bafilomycin A1. Data (mean ± SEM) are representative of at least three independent experiments. Statistical significance was determined by one-way analysis of variance followed by Bonferroni post-hoc test. ^#^ *p* < 0.05 compared between groups. * *p* < 0.05 compared to control.

Chronic rotenone exposure, which produces nigral degeneration and movement disturbances, leads to weight loss and reduced tyrosine hydroxylase (TH)-positive cells in the rat striatum and substantia nigra, ultimately resulting in animal death (Figure S1). Rotenone also induces mitochondrial damage andautophagosome formationin the rat striatum (Figure S2). Based on these observations, we used a rotenone-induced cellular model of PD to further clarify the potential protective effect of autophagy on neuronal survival. We first examined the dose response of rotenone in the human dopaminergic cell line, SH-SY5Y, and found that the median lethal dose (LD_50_) of rotenone was 20 μM after a 24 h exposure (Figure 1A). We then determined that 20 μM resveratrol was effective in preventing rotenone-induced cell death (Figure 1B), thus this concentration was used throughout the following experiments. As autophagosomal structures developed in the rotenone PD animal model (Figure S2C), we hypothesized that autophagy may play a role in resveratrol-mediated neuronal survival. As shown in Figure 1C,D, resveratrol rescued rotenone-induced cell death and this effect was prevented by bafilomycin A1, a specific inhibitor of vacuolar-type H^+^-ATPase that blocks autophagosome-lysosome fusion. Treatment with bafilomycin A1 alone significantly induced accumulation of the autophagosomal marker LC3-II (microtubule-associated protein 1A/1B-light chain 3), but had no significant effect on cell death (Figure S3). This suggests that the pro-survival effect of resveratrol against rotenone-induced cell death is associated with autophagy. In addition, TUNEL assays and western blot analysis for active caspase-3, an apoptosis marker, suggest that resveratrol-induced autophagy may play a role in resveratrol-mediated protection against apoptosis (Figure 2).

### 2.2. Resveratrol Increases Rotenone-Induced Autophagosome Formation

**Figure 3.**
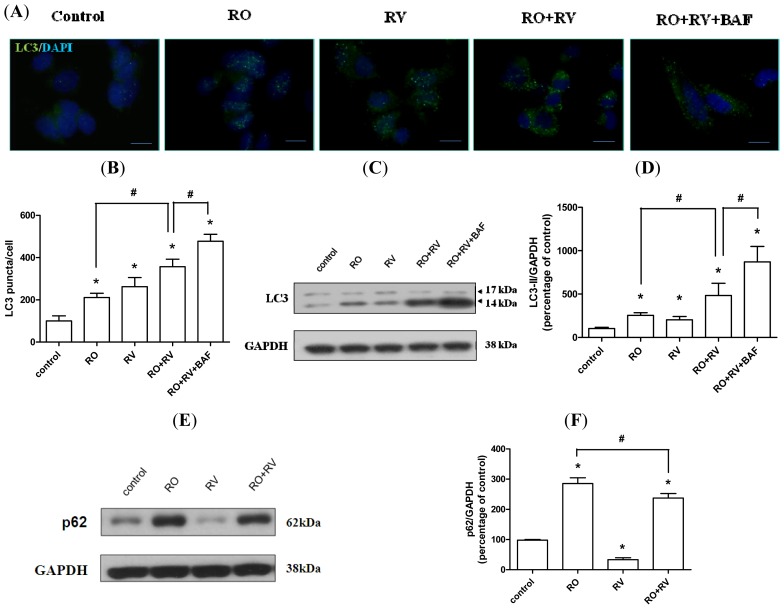
Resveratrol prevents rotenone-mediated inhibition of autophagy. SH-SY5Y cells were pretreated with/without 20 μM resveratrol for 24 h, then treated with/without 20 μM rotenone in the presence/absence of 10 nM bafilomycin A1 for the next 24 h. (**A**,**B**) LC3 puncta (green dots) formation was examined by fluorescence microscopy and quantified using Image J. Nuclei were observed by DAPI staining (blue). Scale bar, 10 μm. The levels of LC3 (**C**,**D**) and p62 (**E**,**F**) were determined by immunoblotting followed by quantitative analysis using Image J. Data were normalized to glyceraldehyde-3-phosphate dehydrogenase (GADPH) levels. RO, rotenone; RV, resveratrol; BAF, bafilomycin A1. Data (mean ± SEM) are representative of at least three independent experiments. Statistical significance was determined by one-way analysis of variance followed by Bonferroni post-hoc test. ^#^ *p* < 0.05 compared between groups. * *p* < 0.05 compared to control.

Microtubule-associated protein 1A/1B-light chain 3 (LC3) is an autophagosome marker. The conversion of the soluble form (LC3-I) in the cytosol to the autophagosome-bound form (LC3-II) indicates an increase in autophagosome formation, which is typically proportional to the increase in autophagic flux [40,41]. Moreover, LC3 puncta that represent autophagosome formation are often observed in cells during autophagic activation [41]. In this study, more LC3 puncta were observed in cells treated with either rotenone or resveratrol than in control cells. Rotenone combined with resveratrol induced more LC3 puncta than rotenone alone (Figure 3A). Bafilomycin A1 blocks late-stage autophagolysomal formation, and thus, as expected, more LC3 puncta were observed in cells treated with a combination of bafilomycin A1, rotenone and resveratrol than in cells treated with a combination of rotenone and resveratrol (Figure 3A,B). Similar results were obtained for LC3-II level because both rotenone and resveratrol increased LC3-II level. LC3-II level was also higher in cells treated with a combination of rotenone plus resveratrol than in cells treated with either rotenone or resveratrol alone. Furthermore, the highest levels of LC3-II were observed in cells treated with a combination of rotenone, resveratrol, and bafilomycin A1 (Figure 3C,D). Increased LC3-II levels may not directly correspond to increased autophagic flux, but could instead indicate autophagosomal accumulation due to inhibition of autophagic flux at a lysosomal level. Thus, we measured the expression pattern of another autophagic marker, p62, which serves to link ubiquitinated proteins to the autophagic machinery via LC3 [42]. During the late stages of autophagy, p62 and p62-bound polyubiquitinated proteins that are incorporated into the autophagosome are degraded in autolysosomes. Accordingly, p62 serves as an indicator of autophagic degradation [43]. As shown in Figure 3E,F, rotenone treatment increased p62 levels, whereas resveratrol decreased p62 levels. Notably, p62 levels were lower in cells treated with rotenone plus resveratrol than in cells treated with rotenone alone (Figure 3E,F), suggesting that rotenone inhibits autophagic degradation, while resveratrol induces degradation to promote cell survival. In addition, bafilomycin A1 reversed the deceased p62 level caused by resveratrol whereas bafilomycin A1 did not affect rotenone-induced p62 level (Figure S4). This data verified that bafilomycin A1-sensitive autophagic flux is augmented by resveratrol while is suppressed by rotenone. Together, these results imply that resveratrol promotes autophagosome formation and facilitates autophagic protein degradation.

### 2.3. Resveratrol Prevents Rotenone-Mediated Inhibition of HO-1 Expression

**Figure 4.**
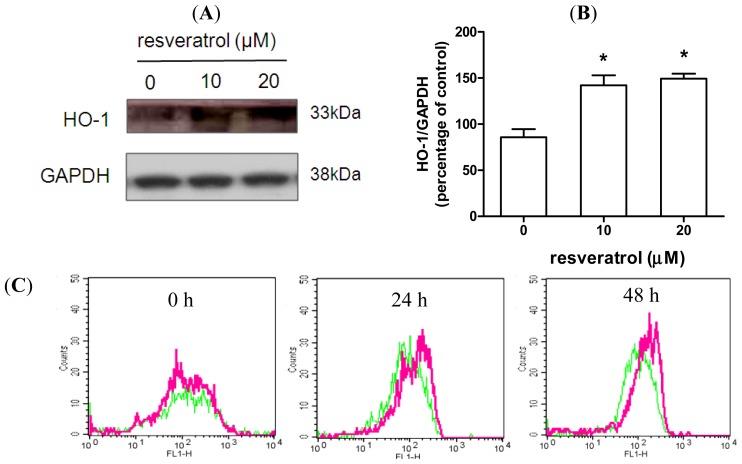

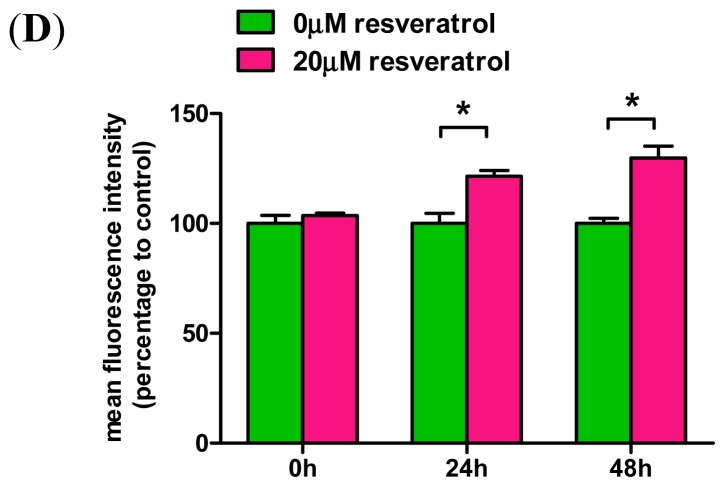
Resveratrol increases HO-1 expression. (**A**,**B**) SH-SY5Y cells were treated with 0–20 μM resveratrol for 48 h. HO-1 expression was determined by immunoblotting followed by quantitative analysis using Image J. Data were normalized to glyceraldehyde-3-phosphate dehydrogenase (GADPH) levels. Data (mean ± SEM) are representative of at least three independent experiments. * *p* <0.05 compared to untreated control; (**C**,**D**) Cells were treated with/without 20 μM resveratrol for 0, 24, or 48 h, followed by fixation and permeabilization. HO-1 levels were determined by flow cytometry. Data (mean ± SEM) are representative of at least three independent experiments and are expressed as the percentage of mean fluorescence intensity (MFI) relative to control at each time point. Statistical significance was determined by one-way analysis of variance followed by Bonferroni post-hoc test. * *p* < 0.05 compared to control.

**Figure 5.**
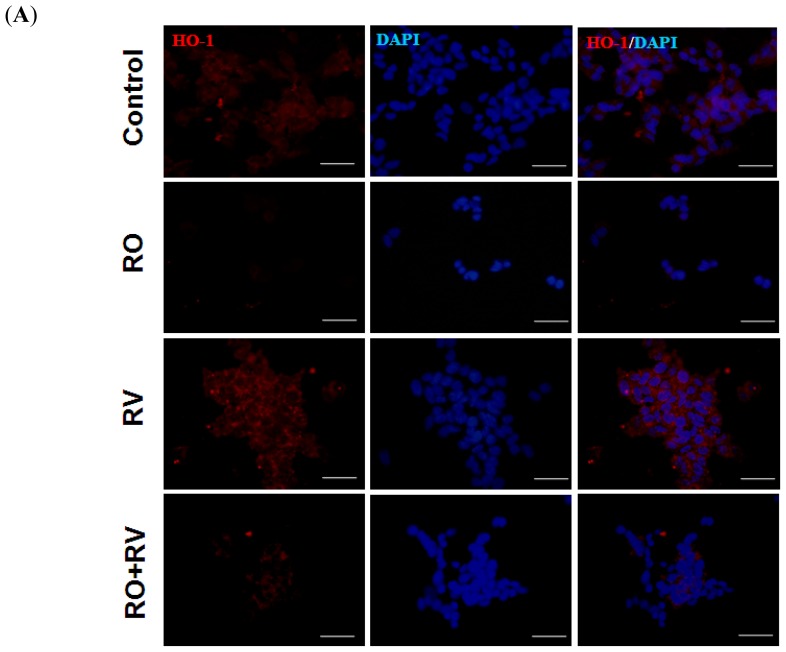

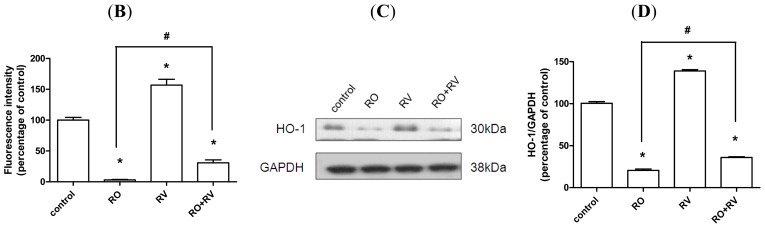
Rotenone-mediated inhibition of HO-1 expression is prevented by resveratrol. SH-SY5Y cells were pretreated with/without 20 μM resveratrol for 24 h then treated with/without 20 μM rotenone for the next 24 h. (**A**,**B**) HO-1 expression (red) was determined by fluorescence microscopy and quantified using Image J. Nuclei were observed by DAPI (blue) staining. Scale bar, 50 μm; (**C**,**D**) HO-1 expression was determined by immunoblotting followed by quantitative analysis using Image J. Data were normalized to glyceraldehyde-3-phosphate dehydrogenase (GADPH) levels. RO, rotenone; RV, resveratrol. Data (mean ± SEM) are representative of at least three independent experiments. Statistical significance was determined by one-way analysis of variance followed by Bonferroni post-hoc test. ^#^ *p* < 0.05 compared between groups. * *p* < 0.05 compared to control.

HO-1, an inducible isoform of the HO enzyme, acts to degrade intracellular heme to form free iron, carbon monoxide and biliverdin. It confers protection against exogenous stress and inhibits cell apoptosis in a number of tissue injury models [33]. To determine whether HO-1 is associated with the neuroprotective effect of resveratrol, SH-SY5Y cells were treated with various doses of resveratrol over a 48 h period. Both 10 and 20 μM resveratrol induced HO-1 expression (Figure 4A,B). The dose of resveratrol required to induce HO-1 was similar to the dose required to prevent rotenone-induced cell death (Figure 1B). Resveratrol (20 μM) increased HO-1 expression throughout the 24 to 48 h period (Figure 4C,D). Next, the role of HO-1 in resveratrol-mediated protection against rotenone intoxication was investigated. As shown in Figure 5, rotenone reduced HO-1 expression, whereas resveratrol increased HO-1 expression. Furthermore, resveratrol partially prevented the effect of rotenone on HO-1 expression, in both the immunofluorescence assay and the immunoblotting assay (Figure 5). In neuronal cells, the protective effects of HO-1 are associated with suppression of oxidative stress [44,45]. Thus, we examined the effect of resveratrol on intracellular levels of reactive oxygen species (ROS). As shown in Figure S5, rotenone, but not resveratrol, induced ROS generation. Notably, resveratrol prevented rotenone-induced ROS generation (Figure S4). Together, these data suggest that the effects of rotenone and resveratrol are, at least in part, mediated through HO-1 expression.

### 2.4. Resveratrol Prevents Rotenone-Induced Neuronal Death through HO-1 Dependent Autophagy

**Figure 6.**
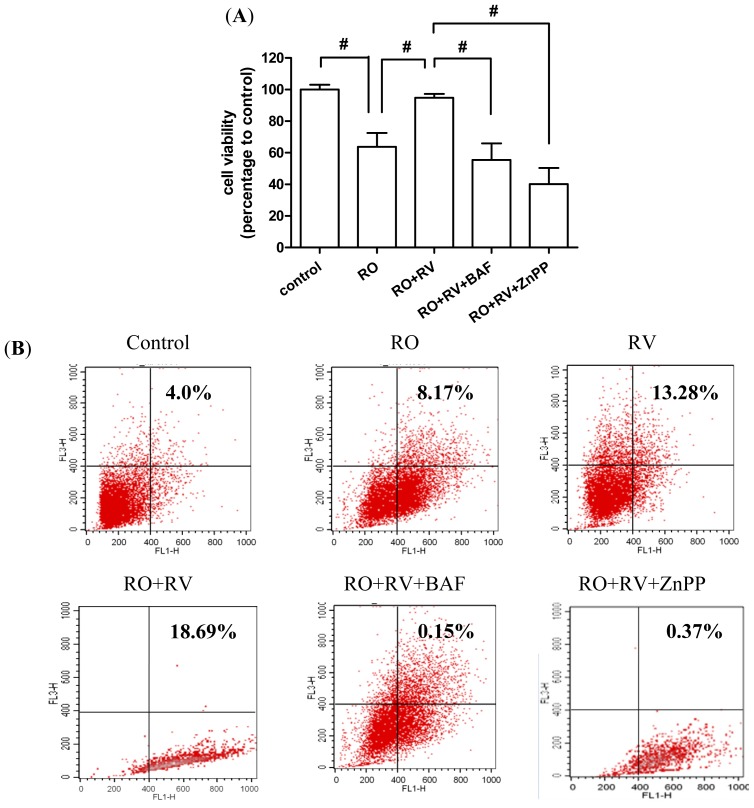

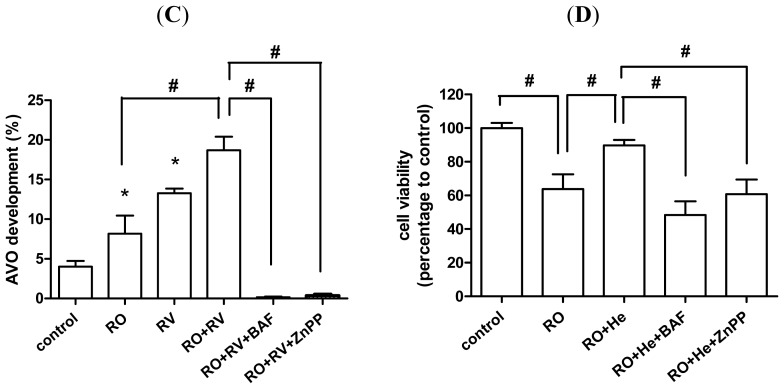
The protective effects of resveratrol are mediated by HO-1-dependent autophagy. (**A**,**B**,**C**) SH-SY5Y cells were pretreated with/without 20 μM resveratrol for 24 h then treated with/without 20 μM rotenone in the presence/absence of 10 nM bafilomycin A1 or 10 μM ZnPP for the next 24 h. (**A**) Cell viability was determined by WST-8 assay; (**B**,**C**) Acidic vesicular organelles (AVOs) stained by acridine orange were analyzed by cytometry. AVO formation was defined as the percentage of cells in the top grid of each panel; (**D**) SH-SY5Y cells were pretreated with/without 5 μM hemin for 24 h then treated with/without 20 μM rotenone in the presence/absence of 10 nM bafilomycin A1 or 10 μM ZnPP for the next 24 h. Cell viability was determined by WST-8 assay. RO, rotenone; RV, resveratrol; BAF, bafilomycin A1; ZnPP, zinc protoporphyrin-IX; He, hemin. Data (mean ± SEM) are representative of at least three independent experiments. Statistical significance was determined by one-way analysis of variance followed by Bonferroni post-hoc test. ^#^ *p* < 0.05 compared between groups. * *p* < 0.05 compared to control.

To further confirm the role of HO-1 in resveratrol-mediated protection against rotenone-induced apoptosis, a chemical inhibitor of HO-1, zinc protoporphyrin (ZnPP), was used to investigate the effects of resveratrol. Resveratrol effects on cell viability were eliminated by ZnPP treatment (Figure 6A). We then investigated whether HO-1 induces autophagy by examining acidic vesicular organelle (AVO) formation using acridine orange, as previously reported [46]. In contrast to the bafilomycin A1-induced increase in LC3-II level (Figures 3C,D), bafilomycin A1 treatment markedly suppressed AVO development (Figure 6B). This further supports the observation that AVO formation reflects autophagolysome development, since bafilomycin A1 prevents autophagosome and lysosome fusion. Both rotenone and resveratrol treatment induced AVO formation (Figures 6B,C). Notably, AVO formation was > 2-fold higher in cells treated with rotenone plus resveratrol than in cells treated with rotenone alone, suggesting that resveratrol increases autophagic flux (Figures 6B,C). Importantly, the HO-1 inhibitor ZnPP markedly prevented AVO formation (Figure 6B,C). To further verify that HO-1 has a protective action in rotenone-induced cell death, experiments were performed using hemin, a pharmacological inducer of HO-1 expression. As shown in Figure 6D, hemin prevented rotenone-induced cell death. This pro-survival effect of hemin was abrogated by ZnPP and bafilomycin A1 (Figure 6D), suggesting that hemin increases resistance to neurotoxins through an autophagy-dependent pathway. Serving as experimental control, hemin or ZnPP alone did not affect cell viability (Figure S6A). In addition, hemin exhibited identical effect to resveratrol in enhancing AVO formation (Figure S6B,C), supporting the hypothesis that resveratrol increases HO-1 level to induce autophagy. Taken together, these results demonstrate that the protective effects of resveratrol on rotenone-induced neuronal cell death are mediated by HO-1 and sequential induction of autophagy.

### 2.5. Discussion

Autophagic flux is a dynamic process that includes initiation, elongation, maturation and degradation. Recently, autophagy dysfunction has been linked to PD development in humans and in mice with a conditional knockout of *Atg7* [47]. Herein, we provide evidence that resveratrol induces HO-1 to augment autophagic flux and thus prevent rotenone-induced neuronal apoptosis. This is a novel insight into the cellular mechanisms of resveratrol in preventing neurodegeneration, and provides potential downstream targets for future therapeutic strategies.

Autophagy is active at a basal level in most cells in the body, and this probably reflects its role in regulating the turnover of long-lived proteins and degradation of damaged structures [48]. In particular, selective autophagy of mitochondria can limit ROS production and prevent the release of cytochrome c to the cytosol, thereby acting as a protective mechanism against apoptosis [49,50]. We recently demonstrated that resveratrol induces anti-apoptotic signaling, mitochondrial biogenesis and upregulation of LC3-II in rats with cholestasis-induced liver injury [27]. Notably, in SH-SY5Y cells, rapamycin [51,52] and deferoxamine [53] increase autophagy to protect against rotenone-induced apoptosis, whereas 3-methyladenine and bafilomycin A1 inhibit autophagy and increase rotenone-induced apoptosis [51]. In the present study, we used an *in vitro* PD model to verify that the neuroprotective effects of resveratrol are mediated through increased autophagy. The observed resveratrol-mediated increase in autophagic flux likely acts to degrade mitochondria damaged by the mitochondrial complex I inhibitor rotenone, prevents the release of pro-apoptotic molecules and aids cellular survival. Similar results were obtained in a recent study that demonstrated resveratrol-mediated neuroprotection through autophagy [54]. By contrast, despite using higher doses than the present study, resveratrol was reported to have no protective effects on rotenone-induced apoptosis in SH-SY5Y cells [55]. However, cells were pretreated with resveratrol for 1 h prior to rotenone exposure [55], as opposed to 24 h in the present study. Thus, a sufficient pretreatment time is required to enable expression of critical neuroprotective and anti-apoptotic genes. In support of this hypothesis, we demonstrated that increased HO-1 expression in response to resveratrol confers neuroprotective effects in an autophagy-dependent manner.

Rotenone produces oxidative damage, endoplasmic-reticulum stress and cell apoptosis, together with the accumulation of α-synuclein and ubiquitin *in vitro* [56]. *In vivo*, chronic exposure to rotenone induces cytoplasmic inclusions similar to Lewy bodies [57]. Furthermore, we demonstrated that rotenone induces apoptosis in dopaminergic neurons of the rat substantia nigra and striatum [58]. In the present study, we show loss of dopaminergic neurons and oxidative damage in mitochondria, together with autophagosomal development *in vivo* (Figures S1 and S2) and impaired autophagic flux *in vitro* (Figure 5), implying the occurrence of this pathological accumulation. Autophagy-dependent p62 degradation is inhibited by rotenone *in vitro*. Hence, rotenone treatment is associated with inadequate clearance of unfolded proteins and organelle damage. In support of our results, Mader *et al.* observed inhibitory-autophagic flux in a rotenone-induced PD model *in vitro* [59]. Although autophagosome formation is typically proportional to autophagic flux, in the present study, bafilomycin A1 further potentiated rotenone and resveratrol-mediated increases in LC3 puncta and LC3-II level. However, bafilomycin A1 also blocked the anti-apoptotic effect of resveratrol (Figure 1). Thus, resveratrol likely increases autophagic flux, not solely through increased autophagosomal development. In accordance with this view, resveratrol, which promotes LC3-II level and clearance of p62, facilitated autophagic flux (Figure 3), prevented rotenone-mediated inhibition of autophagy and attenuated apoptosis (Figures 1 and 2). Autophagy is dependent upon autophagolysosome development [31,32] and the subsequent involvement of proteolytic enzymes in the lysosome, including cathepsin L, B and D [60,61]. Notably, resveratrol induces autophagolysosomal development [29] and cathepsin L [28] and D [30] activity. In the present study, bafilomycin A1 was used to inhibit autophagosome-lysosome fusion, which prevented resveratrol-induced inhibition of cell death and resveratrol-induced AVO formation (Figure 6). This suggests that resveratrol attenuates neurotoxicity through increased autophagolysosome formation. In addition, resveratrol alone caused reduction in cell count (Figure 1), which may indicate that resveratrol suppresses neuronal cell proliferation or growth. Our data demonstrated that resveratrol alone could augment autophagy (Figures 3 and 6). Induction of autophagy is associated with growth inhibition [62]. Therefore, the possibility that resveratrol inhibits cell growth through induction of autophagy cannot be excluded and it should be cautions when studying the effect of resveratrol *in vivo*.

As a phase 2 enzyme, HO-1 is induced by multiple forms of chemical and physical cellular stress. It acts as a general marker of cellular oxidative stress, and confers cytoprotection in numerous models of oxidative injury [33]. HO-1 also protects against neuronal damage in a model of ischemic stroke [63,64]. Furthermore, local injection of adenovirus-expressing HO-1 protects against dopaminergic cell death in a rat PD model induced by 1-methyl-4-phenylpyridinium (MPP+), a mitochondrial complex I inhibitor [65]. These reports indicate that HO-1 promotes neuronal survival during both oxidative stress and following treatment with a mitochondrial toxin. The present data support this hypothesis because resveratrol increased HO-1 expression and prevented rotenone-induced neuronal death. Furthermore, resveratrol attenuated rotenone-induced intracellular ROS generation (Figure S4), which also corresponded to increased HO-1 expression. On the other hand, treatment with resveratrol alone had no effect on ROS generation (Figure S4) while increased HO-1 expression (Figures 4 and 5). The result may explain why the resveratrol-mediated decrease in cell viability (Figure 1D) had no effect on apoptosis (Figure 2) in our model. In addition, HO-1-dependent induction of autophagy may provide a defense mechanism in several disease models. Hepatocyte death induced by experimental sepsis *in vivo* or lipopolysaccharide (LPS) *in vitro* is exacerbated by either inhibition or siRNA knockdown of HO-1, suggesting a pro-survival induction of autophagy via HO-1 action [66]. The same group also demonstrated that in macrophages, LPS induces the release of proinflammatory cytokines together with a concomitant increase in autophagy via a toll-like receptor-4 (TLR4)/HO-1-dependent pathway [67]. In an acute kidney injury model induced by cisplatin, HO-1 and LC3-II are up-regulated in proximal tubular epithelial cells [68]. By contrast, proximal tubular epithelial cells of HO-1-knockout mice demonstrate significant apoptosis and impaired induction in autophagy [68]. These reports demonstrate the critical role of HO-1 in regulating autophagy, which is of particular importance in the present study where HO-1 action was associated with resveratrol-mediated autophagic flux in dopaminergic cells.

## 3. Experimental section

### 3.1. Animals and Experimental Model of Parkinson’s Disease

Experiments were performed on specific pathogen-free adult male Lewis rats (300–350 g) obtained from the Experimental Animal Center of the National Science Council, Taiwan. Rats were housed in a temperature-controlled room (24–25 °C) with a 12 h light-dark (08:00–20:00) cycle. Standard laboratory rat chow and tap water were available *ad libitum*. The experimental animal model of PD was induced by subcutaneous administration of rotenone, a specific inhibitor of mitochondrial complex I, as described previously [59].

### 3.2. Cell Culture

Human neuroblastoma SH-SY5Y cells (BCRC 67018; Bioresource Collection and Research Center, Taiwan) were grown in DMEM/F12 medium containing 10% fetal bovine serum (FBS), 1× GlutaMAX, 100 U/mL penicillin and 100 μg/mL streptomycin (all from Gibco, Carlsbad, CA, USA) at 37 °C with 5% CO_2_. Low passage number (below 15 to 20) cells were used for all experiments. Stocks of rotenone, resveratrol, bafilomycin A1, and ZnPP (Sigma, St. Louis, MO, USA) were dissolved in dimethyl sulfoxide (DMSO) and stored at −20 °C. Drug stocks were prepared for single use to avoid repeated free-thaw cycles. Cells were pretreated with resveratrol for 24 h prior to exposure to rotenone in the presence/absence of bafilomycin A1 or ZnPP.

### 3.3. Isolation of Striatal Mitochondria and Detection of Oxidized Proteins

Following exposure to rotenone or DMSO/polyethylene glycol (as a control) for 28 days, rats were anesthetized, their brains were removed, and their whole striata isolated. Tissue samples were homogenized in 10 mL buffer A (320 mM sucrose, 5 mM Tris, 2 mM EGTA, pH 7.4, at 4 °C) with 5 strokes of a Teflon Dounce homogenizer. Samples were centrifuged for 3 min at 2000 × *g* to remove nuclei and tissue particles. Supernatants were then collected and centrifuged for 10 min at 12,000× *g* to pellet mitochondria and synaptosomes. The crude pellet was resuspended in 10 mL of buffer A with the addition of 0.02% *w*/*v* of digitonin to disrupt synaptosomal membranes and release trapped mitochondria. The resuspended pellet was then centrifuged for 10 min at 12,000× *g* to pellet mitochondria, which were then resuspended in 100 μL of buffer A. Protein content was determined by BCA assay (Pierce, Rockford, IL, USA).

Proteins were extracted from the mitochondrial fraction of the striatal samples by using a commercial kit (Active Motif, Carlsbad, CA, USA). Oxidized proteins were assayed by immunodetection of carbonyl groups, which is a hallmark of the oxidation status of proteins. A protein oxidation detection kit (Chemicon, Temecula, CA, USA) was used to detect oxidized proteins. Briefly, proteins were extracted from striatal tissue at various time points after exposure to rotenone, and were reacted with 2,4-dinitrophenylhydrazine and derivatized to 2,4-dinitrophenylhydrazone (DNP-hydrazone). The DNP-derivatized protein samples were separated on a 15% SDS-polyacrylamide gel then detected by western blotting. Blots were incubated with a rabbit anti-DNP primary antibody followed by incubation with a horseradish peroxidase-conjugated goat anti-rabbit IgG secondary antibody according to manufacturers’ instructions.

### 3.4. Immunohistochemical Staining

To evaluate dopaminergic neuronal loss in the striatum and substantia nigra following chronic rotenone intoxication, immunohistochemical staining for tyrosine hydroxylase was performed as previously described [59]. Briefly, rat brains were embedded in tissue-freezing medium (Sakura Finetek, Torrance, CA, USA), serially sectioned in the coronal plane throughout the rostral-caudal extent of striatum and substantia nigra at 7 μm intervals on a cryostat, and mounted on SuperfrostPlus slides (Fisher Scientific, Waltham, MA, USA). Sections were permeabilized with 0.3% Triton X-100 and 10% horse serum in 0.01 M phosphate-buffered saline (PBS) for 20 min, prior to incubation with primary antibodies. A mouse monoclonal antibody against tyrosine hydroxylase (Chemicon Temecula, CA, USA) was applied to the sections overnight at 4 °C. The following day the brain sections were incubated with a secondary biotinylated goat anti-mouse immunoglobulin G (IgG) (Vector Laboratories, Burlingame, CA, USA).

### 3.5. Electron Microscopy

Rat striata were removed and processed for electron microscopy as previously described [69]. Briefly, tissue samples were diced and submerged in 4% glutaraldehyde (0.1 M sodium cacodylate buffer, pH 7.2). Tissues were postfixed with osmium, and stained with uranyl acetate. After dehydration, each specimen was embedded by infiltration in Spurr’s medium. Tissue blocks were trimmed, and 90 nm thick sections were cut, poststained with uranylacetate and lead citrate, mounted on coated 300 mesh grids, and examined using a JEOL JEM-1230 (Tokyo, Japan) electron microscope. For measurement of autophagosome number, 15 randomly selected striatal areas per animal, which included large neuronal-like nuclei covering about one fourth of the visible image, were photographed at ×8000 magnification and counted according to our previous work [69], with minor modifications.

### 3.6. Cell-Survival Assay

For cell-viability assays, 7.5 × 10^3^ SH-SY5Y cells were seeded in 96-well dishes. Following treatment, bright field images were examined for changes in cell morphology and viability. Cells were trypsinized, stained with trypan blue and counted using a hemocytometer. In some experiments, cell survival was determined using Cell Counting Kit 8 (Wako, Richmond, VA, USA) with WST-8 (2-(2-methoxy-4-nitrophenyl)-3-(4-nitrophenyl)-5-(2,4-disulfophenyl)-2-H-tetrazolium, monosodium salt) as the substrate. WST-8 is reduced by dehydrogenase to form formazan. The concentration of formazan produced is proportional to the number of living cells. Formazan was assayed by spectrophotometry at OD450. For the terminal deoxynucleotidyl transferase dUTP nick end labeling (TUNEL) fluorescence assay, 1 × 10^5^ SH-SY5Ycells were harvested and seeded onto cover slips in 24-well dishes. After treatment, cells were fixed in 1% formaldehyde, permeabilized in 1% Triton X-100 and 1% sodium citrate for 2 min at 4 °C, and stained using a TUNEL kit (Millipore, Billerica, MA, USA). Cover slips were mounted in media containing 1 μg/mL 4′,6-diamidino-2-phenylindole (DAPI) (Invitrogen, Carlsbad, CA, USA). TUNEL-positive cells were examined by fluorescence microscopy.

### 3.7. Immunoblotting

Cells were washed with PBS and lysed with Protein Extraction Buffer (Intron Biotechnology, Gyeonggi-do, Korea) on ice for 20 min. Solubilized cells were centrifuged at 10,000× *g* for 10 min and the supernatant was collected. Protein concentrations were determined by Bradford assay. Protein samples (20–40 μg) were loaded onto a 12%–15% SDS-polyacrylamide gel, separated by electrophoresis, then transferred to PVDF membranes. Membranes were blocked with 10% fat-free milk in Tris-buffered saline (TBS) and 0.05% Tween-20 (TBST) at room temperature for 1 h. Membranes were then probed with primary antibodies against target proteins overnight at 4 °C. After three washes in TBST, membranes were incubated with a secondary antibody conjugated to horseradish peroxidase (HRP) (Genetax, Irvine, CA, USA) diluted at 1:5000 in TBST for 1 h at room temperature. Proteins were detected by enhanced chemiluminescence (ECL) (Millipore, Billerica, MA, USA) using X-ray films. Primary antibodies against cleaved caspase-3, HO-1, p62 and LC3 were purchased from Abcam (Abcam plc, Cambridge, UK).

### 3.8. Immunocytochemistry

SH-SY5Y cells grown on coverslips were washed twice with PBS and fixed with 4% paraformaldehyde in PBS for 10 min at room temperature. Cells were then washed twice with PBS, permeabilized with 0.1% Triton X-100 in PBS and were again washed twice with PBS. After blocking with 10% fat-free milk in PBS for 30 min at room temperature, cells were incubated with primary antibodies against LC3 or HO-1 (Abcam plc, Cambridge, UK) for 1 h at room temperature. Cells were washed four times with PBS followed by incubation with secondary antibodies (Abcam plc, Cambridge, UK) for 1 h at room temperature. Subsequently, cells were washed three times with PBS and mounted with media containing DAPI (Invitrogen, Carlsbad, CA, USA). Samples were analyzed with a fluorescence microscope (Leica, Wetzlar, Germany).

### 3.9. Flow Cytometry

For detection of HO-1 expression, 2 × 10^5^ cells grown in 6-well dishes were fixed in 1% formaldehyde, permeabilized in 1% Triton X-100 for 2 min on ice, and incubated with a FITC-conjugated antibody against HO-1 for 30 min at room temperature. Cells were then washed and resuspended in PBS, followed by analysis using a FACSCalibur cytometer (Becton Dickinson, Franklin Lakes, NJ, USA). For detection of AVO development, 2 × 10^5^ cells grown in 6-well dishes were stained with acridine orange(Sigma, St. Louis, MO, USA) for 15 min, removed from the plate with trypsin-EDTA, and collected in phosphate buffer saline containing 10% fetal bovine serum. Green (510–530 nm; FL1) and red (>650 nm; FL3) fluorescence emission was measured using a FACSCalibur cytometer (Becton Dickinson, Franklin Lakes, NJ, USA) with excitation at 488 nm.

Intracellular ROS generation was determined by flow cytometry, following cell staining with a 6-carboxy-2,7-dichlorodihydrofluorescein diacetate (DCFDA) (Sigma) fluorescent probe. DCFDA is converted by oxidation to highly fluorescent 2′,7′-dichlorodihydrofluorescein (DCF), which was detected at an emission wavelength of 530 nm with excitation at 485 nm. Briefly, cells were washed with PBS twice and stained with 5 μM DCFDA for 30 min at 37 °C. Cell pellets were collected, washed twice with PBS, and then resuspended in PBS. Fluorescence was detected using a FACSCalibur flow cytometer (BD Biosciences, San Jose, CA, USA) and analyzed using CellQuest software (Becton Dickinson, San Jose, CA,USA).

## 4. Conclusions

**Figure 7.**
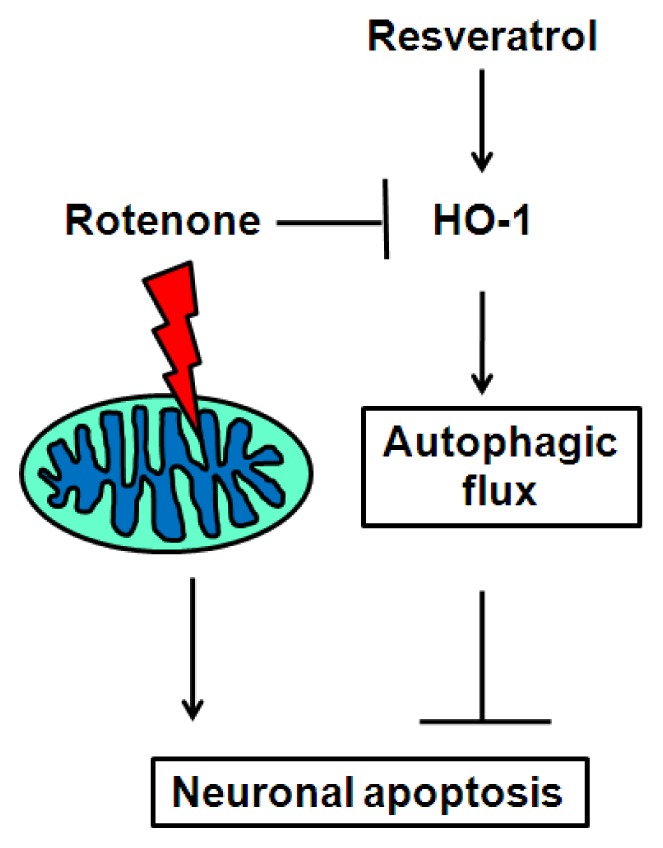
Schematic diagram of the role of HO-1 in mediating resveratrol-induced autophagy and rescue of neurotoxin-induced apoptosis. In a PD model, the mitochondrial complex I inhibitor rotenone causes loss of dopaminergic neurons, mitochondrial damage, autophagosomal formation and reduced autophagic flux. Resveratrol, which augments HO-1 expression in dopaminergic cells, prevents rotenone-induced apoptosis through increased autophagic flux. This scenario may occur when cells or animals suffer from mitochondrial stress and neuronal degeneration, leading to PD. Induction of autophagy by resveratrol or by molecules that induceHO-1 expression may be a therapeutic strategy for neurodegenerative disease.

A hypothetical model that depicts the protective role of resveratrol in inducingHO-1-dependent autophagy is shown in Figure 7. Rotenone suppresses autophagic flux and HO-1 expression, and increases accumulation of autophagic vacuole structures that contribute to dopaminergic neuronal death. Resveratrol promotes HO-1 expression and HO-1-dependent autophagic flux, thereby ultimately preventing rotenone-induced apoptosis. Increased understanding of the pro-survival effects of resveratrol and corresponding mechanisms of neuronal protection may provide therapeutic targets for the treatment of neurodegenerative diseases such as PD.

## Supplementary Information
