# Supplementary material for: Resveratrol Partially Prevents Rotenone-Induced Neurotoxicity in Dopaminergic SH-SY5Y Cells through Induction of Heme Oxygenase-1 Dependent Autophagy
Source: Int J Mol Sci. 2014 Jan 22;15(1):1625–46. doi: 10.3390/ijms15011625 (PMC3907890; doi:10.3390/ijms15011625)
Supplement: Supplementary file 1 [file ijms-15-01625-s001.pdf]

Supplementary Information

Figure S1. Rotenone intoxication reduces body weight and survival rate, and induces neuronal loss in the striatum and substantia nigra of rats. (**A**,**B**) Lewis rats received DMSO/PEG (sham) or 3 mg/kg/day rotenone for 28 days. The survival rate and body weight of six to nine animals per experimental group were examined at the indicated times. Survival curves were compared using the log-rank test. Body weight between groups was compared by independent *t*-test. The statistical significance of the IHC data was determined by one-way analysis of variance followed by Bonferroni post-hoc test. * $p < 0.05$. The expression of tyrosine hydroxylase in the striatum (**C**) and substantia nigra (**D**) were examined by immunohistochemistry (IHC) as described in Experimental sections.

(**A**)

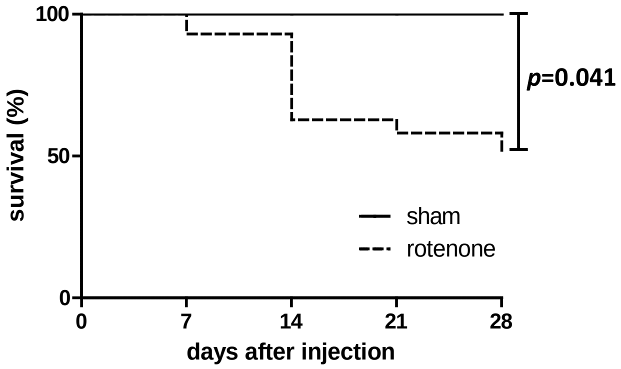

(**B**)

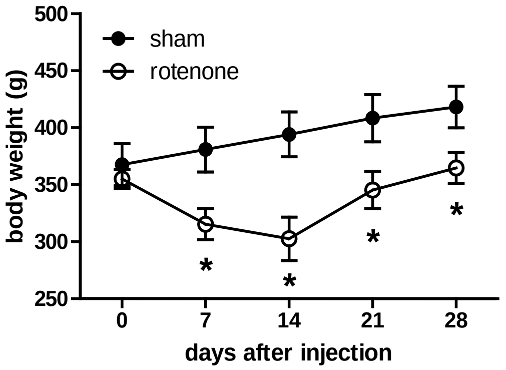

(**C**)

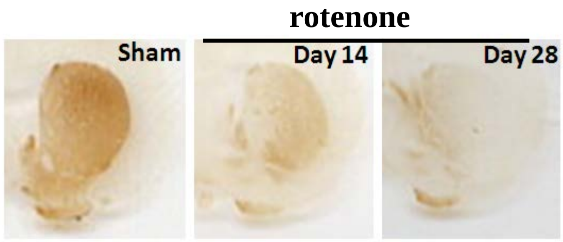

(**D**)

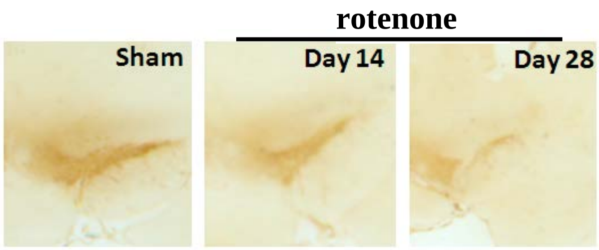

Figure S2. Rotenone induces mitochondrial-oxidative damage and autophagic vacuoles in the rat striatum. (**A**,**B**) Isolated mitochondria from the striatum of Lewis rats that received DMSO/PEG or rotenone were assayed for changes in protein oxidation by immunodetection of carbonyl groups using an anti-DNP antibody. COX IV level examined by immunoblotting was used as loading control. Data (mean ± SEM) are representative of at least three independent experiments. Statistical significance was determined by independent t-test.* $p < 0.05$. (**C**) The striata of rats that received DMSO/PEG or rotenone for 14 days were fixed and subjected to electron microscopy analysis. Black arrowheads (▲) indicate mitochondria with a normal structure. Arrows indicate condensing chromosomes. White arrowheads (Δ) indicate autophagic vacuoles enclosing mitochondria. Asterisks (★) indicate ruptured or swollen mitochondria. Rectangles show an enlarged area in the middle and right panels. Scale bar, 2 μm. (**D**) Quantification of the number of autophagic vacuoles per cell (mean ± SEM) in the electron photomicrographs from three animals per experimental group. Statistical significance was determined by independent t-test.***** $p < 0.05$.

(**A**)

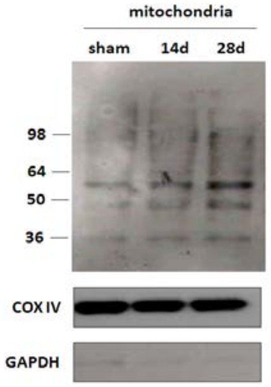

(**B**)

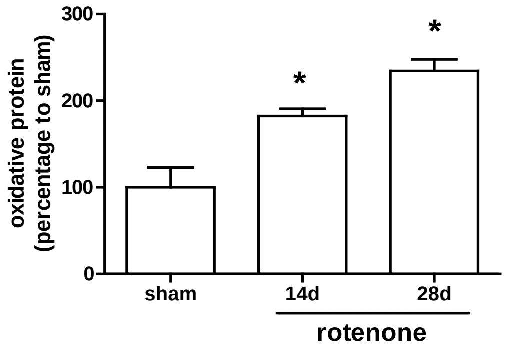

(**C**)

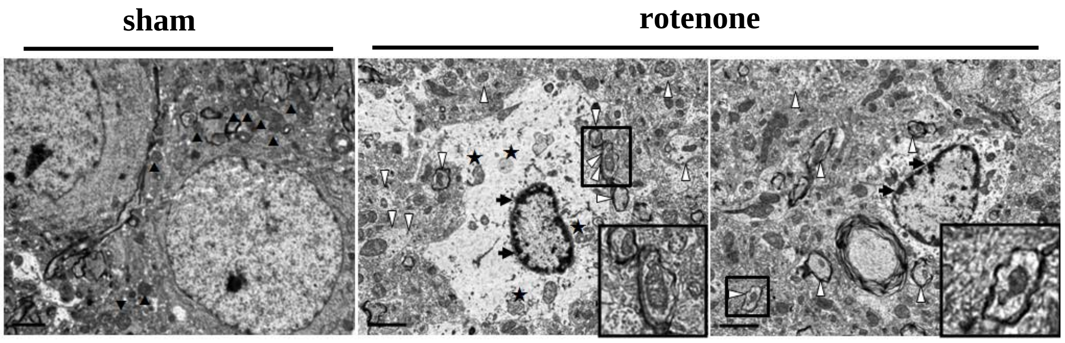

(**D**)

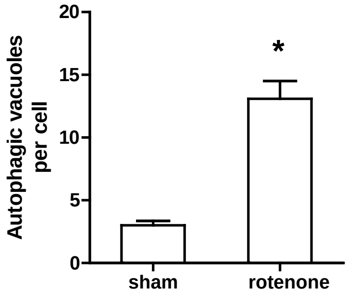

Figure S3. Treatment with 10 nM bafilomycin A1 for 24 h significantly increases LC3-II level but has no apparent effect on HO-1 level, cell morphology and cell viability. (**A**,**B**) The dose response of bafilomycin A1 on LC3 and HO-1 level was examined by immunoblotting. SH-SY5Y cells were grown in the presence of DMSO (0 nM) or various concentrations of bafilomycin A1 for 24 h. Cell viability was determined by light microscopy magnified at 200× (**C**), and by counting trypan blue-stained cells (**D**). Cell counts are presented as a percentage of control. BAF, bafilomycin A1. Data (mean ± SEM) are representative of at least three independent experiments.

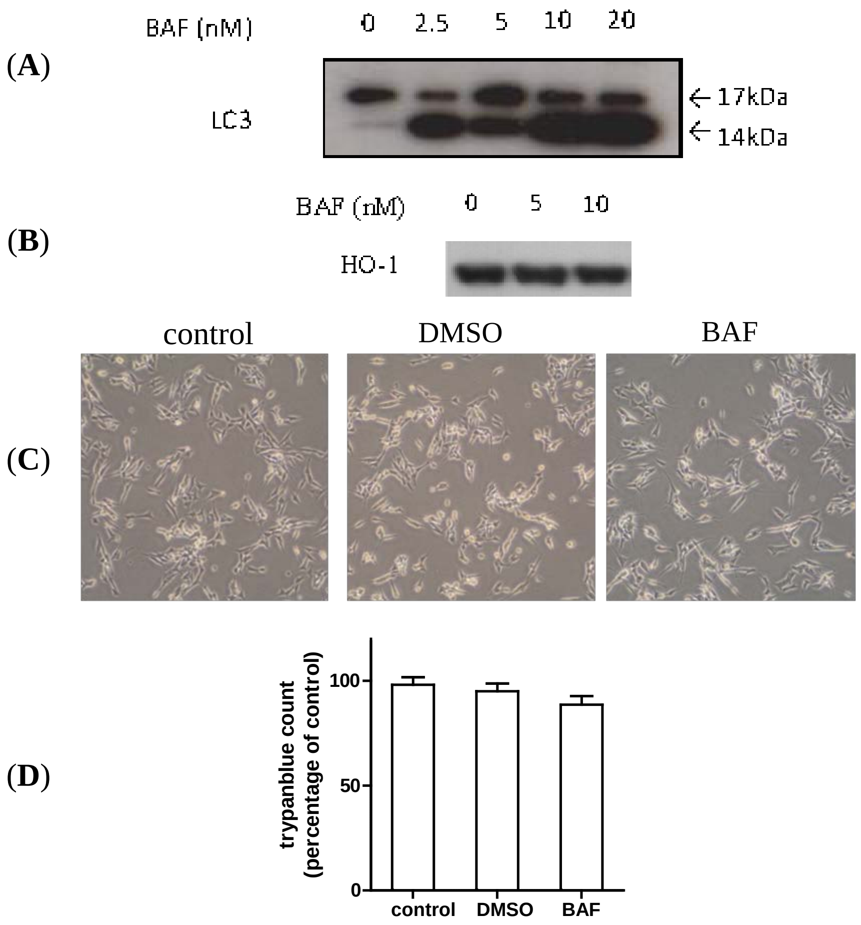

Figure S4. Resveratrol prevents rotenone-mediated inhibition of autophagy. SH-SY5Y cells were pretreated with/without 20 μM resveratrol for 24 h, then treated with/without 20 μM rotenone in the presence of 10 nM bafilomycin A1 or equal volume of DMSO for the next 24 h. (**A**) The levels of LC3 and p62 were determined by immunoblotting followed by quantitative analysis using Image J (**B** and **C**). RO, rotenone; RV, resveratrol; BAF, bafilomycin A1. Data (mean ± SEM) were either shown as ratio of LC3II/I or p63/GADPH, representative of at least three independent experiments. Statistical significance was determined by one-way analysis of variance followed by Bonferroni post-hoc test. # $p < 0.05$ compared between groups. * $p < 0.05$ compared to control.

(**A**)

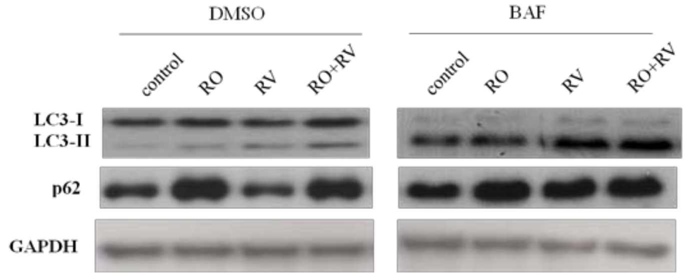

(**B**)

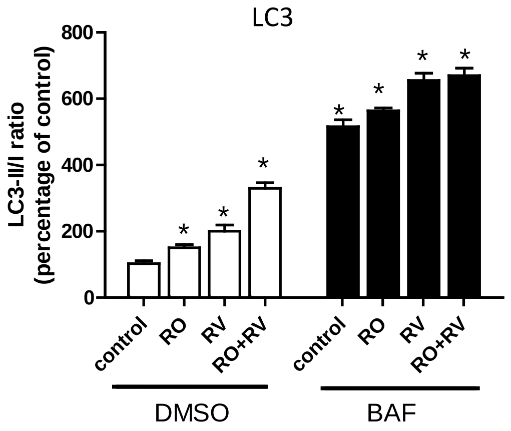

(**C**)

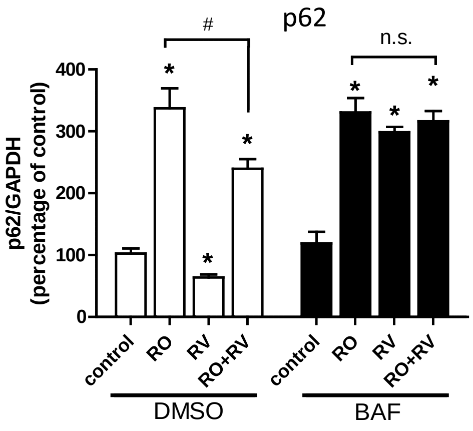

Figure S5. Resveratrol suppresses rotenone-induced ROS generation. SH-SY5Y cells were pretreated with/without 20 μM resveratrol for 24h then treated with/without 20 μM rotenone for the next 24 h. ROS generation was determined by DCF-DA staining and flow cytometry. Data (mean ± SEM) are representative of at least three independent experiments and are expressed as a percentage of control. Statistical significance was determined by one-way analysis of variance followed by Bonferroni post-hoc test. * $p < 0.05$.

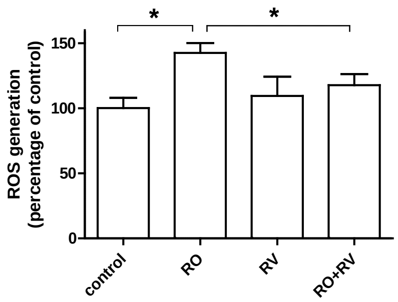

Figure S6. The effects of Hemin and ZnPP alone on cell viability and AVO formation. SH-SY5Y cells were treated with equal volume of DMSO, 5 μM hemin and 10 μM ZnPP for 24 h. (**A**) Cell viability was determined by WST-8 assay. Data (mean ± SEM) presented as a percentage of the control are representative of three independent experiments. (**B**) Acidic vesicular organelles (AVOs) stained by acridine orange were analyzed by cytometry. AVO formation was defined as the percentage of cells in the top grid of each panel. (**C**) Quantitative result of AVO formation. Statistical significance was determined by one-way analysis of variance followed by Bonferroni post-hoc test. * $p < 0.05$ compared to control.

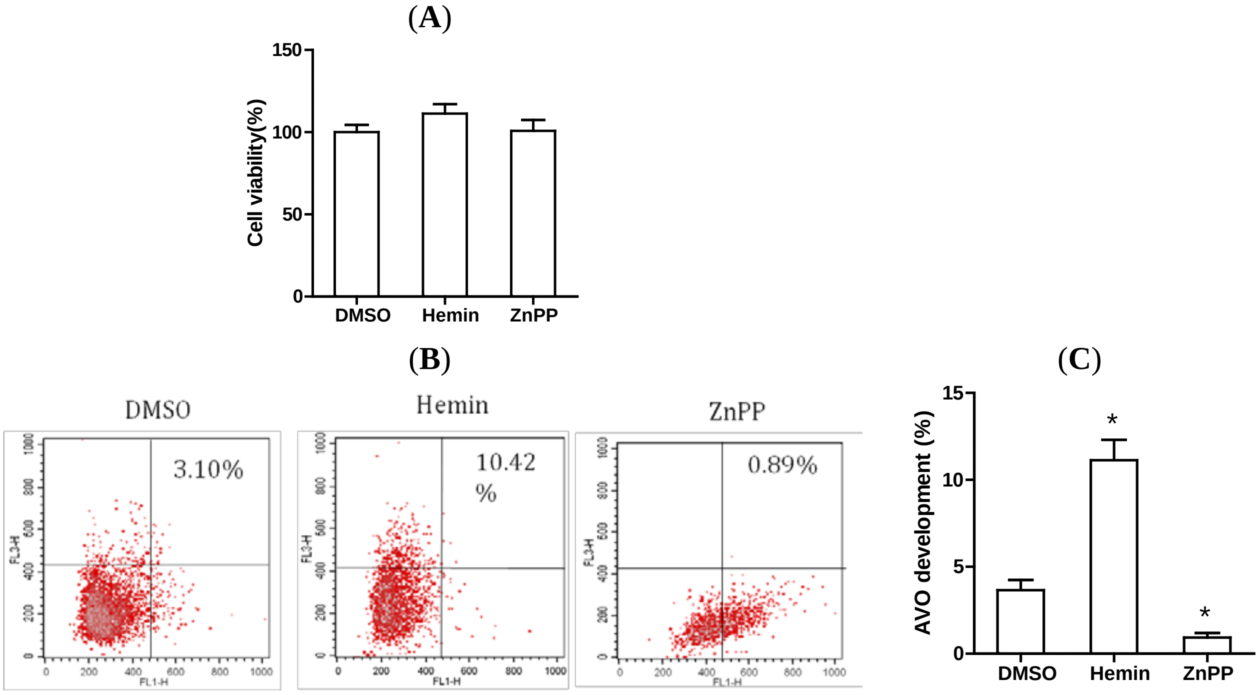

© 2014 by the authors; licensee MDPI, Basel, Switzerland. This article is an open access article distributed under the terms and conditions of the Creative Commons Attribution license (http://creativecommons.org/licenses/by/3.0/).
